# Genome-Wide Identification of RNA Modifications for Spontaneous Coronary Aortic Dissection

**DOI:** 10.3389/fgene.2021.696562

**Published:** 2021-07-02

**Authors:** Tianci Chai, Mengyue Tian, Xiaojie Yang, Zhihuang Qiu, Xinjian Lin, Liangwan Chen

**Affiliations:** ^1^Department of Cardiac Surgery, Fujian Medical University Union Hospital, Fuzhou, China; ^2^Fujian Key Laboratory of Cardio-Thoracic Surgery, Fujian Medical University, Fuzhou, China; ^3^Department of Anesthesiology, Xinyi People’s Hospital, Xuzhou, China; ^4^Key Laboratory of Ministry of Education for Gastrointestinal Cancer, School of Basic Medical Sciences, Fujian Medical University, Fuzhou, China; ^5^Department of Thoracic Surgery, Fujian Medical University Union Hospital, Fuzhou, China

**Keywords:** artery dissection, RNA methylation, genome-wide association study, circulating protein, gene expression

## Abstract

RNA modification plays important roles in many biological processes such as gene expression control. Genetic variants that affect RNA modification may have functional roles in aortic dissection. The aim of this study was to identify RNA modifications related to spontaneous coronary artery dissection (SCAD). We examined the association of RNA modification-associated single-nucleotide polymorphisms (RNAm-SNPs) with SCAD in summary data from a genome-wide association study (GWAS) of European descent (270 SCAD cases and 5,263 controls). Furthermore, we performed expression quantitative loci (eQTL) and protein quantitative loci (pQTL) analyses for the RNAm-SNPs using publicly available data. Functional enrichment and protein–protein interaction analyses were performed for the identified proteins. We found 11,464 unique RNAm-SNPs in the SCAD GWAS dataset, and 519 were nominally associated with SCAD. Nine RNAm-SNPs were associated with SCAD at *p* < 0.001, and among them, seven were N^6^-methyladenosine (m^6^A) methylation-related SNPs, one (rs113664950 in *HLA-DQB1*) was m^7^G-associated SNP, and one [rs580060 in the 3′-UTR of Mitochondrial Ribosomal Protein S21 (*MRPS21*)] was A-to-I modification SNP. The genome-wide significant SNP rs3818978 (SCAD association *p* = 5.74 × 10^–10^) in the 5′-UTR of *MRPS21* was related to m^6^A modification. These nine SNPs all showed eQTL effects, and six of them were associated with circulating protein or metabolite levels. The related protein-coding genes were enriched in specific Gene Ontology (GO) terms such as extracellular space, extracellular region, defense response, lymphocyte migration, receptor binding and cytokine receptor binding, and so on. The present study found the associations between RNAm-SNPs and SCAD. The findings suggested that RNA modification may play functional roles in SCAD.

## Introduction

Aortic dissections (ADs) are complex disorders affecting the aorta. Typically, ADs begin with a tear in the aortic intima, and then blood will intrude into the aortic media and separate the mid-membrane, therefore separating a true from a false lumen. AD is a leading cause of morbidity and mortality. Recently, the Global Burden Disease 2010 project showed that the death rate of AD was increased to 2.78 per 100,000 in 2010 (Sampson et al., 2014a,b). The disease burden increases with age and is more common in men than in women (Sampson et al., 2014a).

Aortic dissection can be inherited, and genetic factors were involved. AD is a heterogeneous disease that can occur in association with syndromes (e.g., Marfan syndrome, Loeys–Dietz aortic aneurysm syndrome) genetically predisposed to AD (Finkbohner et al., 1995; van Karnebeek et al., 2001). Previous studies focused on the syndromes due to variations of *TGFBR2* gene have confirmed the abnormality of the transforming growth factor-beta (TGF-β) pathway regulation as a mechanism contributing to aneurysm formation. Aortic disease in Marfan syndrome is due to the defects in the fibrillin-1 gene at chromosome 15q15-31 (Lee et al., 1991). Genetic studies also have demonstrated that ADs occur in familial aggregates and have identified many genetic loci for this disease (LeMaire et al., 2011; Guo et al., 2016; van ’t Hof et al., 2016; Turley et al., 2020). Identification of functional variants in these genetic loci for AD will increase our understanding of the pathological mechanism of AD.

Recently, genome-wide association studies (GWAS) have identified several genetic loci for spontaneous coronary artery dissection (SCAD), including chromosome 1q21.2 (*ADAMTSL4*), chromosome 6p24.1 (*PHACTR1*), chromosome 12q13.3 (*LRP1*), and chromosome 21q22.11 (*MRP6/KCNE3*) (Saw et al., 2020). This study identified novel genetic factors involved in AD and therefore supported the complex genetic basis of SCAD. However, although some of the identified variants have been shown to affect gene expression, most of their functions have remained unexplained.

To date, more than 170 types of chemical modifications present in RNA molecules have been reported. The RNA modifications are modifiable and are involved in regulations of diverse biological processes in living cells (Malbec et al., 2019). As sufficiently sensitive high-resolution transcriptome-wide techniques developed, several RNA modifications have been widely studied, including N^6^-methyladenosine (m^6^A), m^6^Am, m^1^A, 2′-O-Me, m^5^C, m^5^U, m^7^G, A-to-I, and pseudouridine. Among these known RNA modification types, m^6^A has been discovered as the first example. m^6^A is a type of reversible and widely conserved RNA methylation among eukaryotes. It is known to us because it is important in the regulation of gene expression (Meyer and Jaffrey, 2014) and mRNA stability (Wang et al., 2014) and homeostasis (Edupuganti et al., 2017). It also has been shown to play an important role in the etiology of various diseases (Visvanathan and Somasundaram, 2017). In recent years, researchers have shown that genetic variants would have impacts on all types of RNA modifications by changing the nucleotides at which the modifications occur or RNA sequences around the target sites (Zheng et al., 2018). Therefore, the RNA modification-associated single-nucleotide polymorphisms (RNAm-SNPs) may have impacts on gene expression regulation by influencing RNA modifications and may be important functional variants for SCAD. Currently, the relationship between RNAm-SNPs and SCAD is still unknown. Annotating the functional impacts of RNAm-SNPs on SCAD may help to decipher the pathogenicity mechanisms. This study will present the first effort of evaluating the impacts of the RNAm-SNPs on SCAD genome-wide.

## Materials and Methods

### Determination of RNA Modification-Associated Single-Nucleotide Polymorphisms for Spontaneous Coronary Artery Dissection

In this study, we made use of the novel RNA modification annotations to obtain more functional interpretation of results from the SCAD GWAS (Saw et al., 2020). The GWAS comprised 270 SCAD cases and 5,263 controls of European descent. Summary statistics from the initial GWAS (Saw et al., 2020), which included association *p*-values of 607,778 genotyped variants with SCAD, were downloaded and used as “original data” in the analysis of the present study. The SCAD GWAS summary datasets were publicly available at NHGRI-EBI GWAS Catalog (accession numbers GCST90000582 and GCST90000583).

To identify the RNAm-SNPs in the large amount of SNPs from the GWAS, we obtained a set of RNAm-SNPs in the RMVar database^1^. The RMVar database was an updated version of the m6AVar database. Currently, it contains 1,678,126 RNAm-SNPs for the nine types of RNA modifications (m^6^A, m^6^Am, m^1^A, 2′-O-Me, m^5^C, m^5^U, m^7^G, A-to-I, and pseudouridine), which is four times larger than m6Avar. There are three confidence levels for RNAm-SNPs in the RMVar database, ranging from low confidence to high confidence. RNAm-SNPs derived from single base resolution experiments were classified into high confidence level. Those with medium confidence levels were obtained from MeRIP-Seq experiments. The low-confidence level m^6^A-associated variants were predicted by a CNN model. Based on the annotation of the RNAm-SNP sets, we annotated the GWAS-tested SNPs with RNA modifications in the GWAS summary dataset. Then, RNAm-SNPs that were associated with SCAD were picked out (*p*-values < 0.05 were considered).

### Expression Quantitative Loci Analysis for the Spontaneous Coronary Artery Dissection-Associated RNA Modification-Associated Single-Nucleotide Polymorphisms

The SCAD-associated RNAm-SNPs may affect SCAD risk *via* disturbing RNA modification. Because RNA modification is critical in gene expression regulation, the RNAm-SNPs may act through the regulation of gene expression and therefore may be associated with mRNA expression levels. We therefore performed gene expression quantitative loci (eQTL) analysis to find associations between RNAm-SNPs and mRNA expression levels in different types of cells and tissues. We focused on the association between RNAm-SNPs and the gene in which they are located (*cis*-acting eQTL). Data in the HaploReg browser^2^ were searched (Ward and Kellis, 2012). After RNAm-SNPs with *cis*-acting eQTL effects were found, we tried to identify their functionalities. Transcription regulation functions such as altering protein binding were searched in HaploReg and RegulomeDB^3^. We also looked for eQTL signals in 34 human tissues using data from the GTEx project (v7 release)^4^.

### Protein Quantitative Loci Analysis for the Spontaneous Coronary Artery Dissection-Associated RNA Modification-Associated Single-Nucleotide Polymorphisms

We carried out protein quantitative loci (pQTL) analysis in peripheral blood for the identified RNAm-SNPs to find proteins related to SCAD. The associations between RNAm-SNPs and plasma protein levels were searched in three pQTL studies. The data included, first, the pQTL study that examined the associations between 509,946 SNPs and 1,124 proteins. The summary data were available in the pGWAS Server database^5^ (Suhre et al., 2017). The second pQTL study was the INTERVAL study containing 3,301 individuals of European descent. This study examined the associations between 10.6 million imputed autosomal variants and plasma levels of 2,994 proteins^6^ (Sun et al., 2018). The third pQTL study analyzed 83 proteins measured in 3,394 individuals^7^ (Folkersen et al., 2017). Functional enrichment analyses were performed in the DAVID database, and protein–protein interactions were found in the STRING database.

In addition, we looked for associations between RNAm-SNPs and concentrations of cytokines, lipids, and metabolites. The cytokine pQTL study examined the genome-wide associations for circulating levels of 41 cytokines in 8,293 Finns^8^ (Ahola-Olli et al., 2017). The summary data from a study that examined the associations between genome-wide SNPs and 123 metabolites in blood samples of 24,925 individuals were obtained from http://www.computationalmedicine.fi/data#NMR_GWAS (Kettunen et al., 2016).

## Results

### Spontaneous Coronary Artery Dissection-Associated RNA Modification-Associated Single-Nucleotide Polymorphisms

**FIGURE 1:**
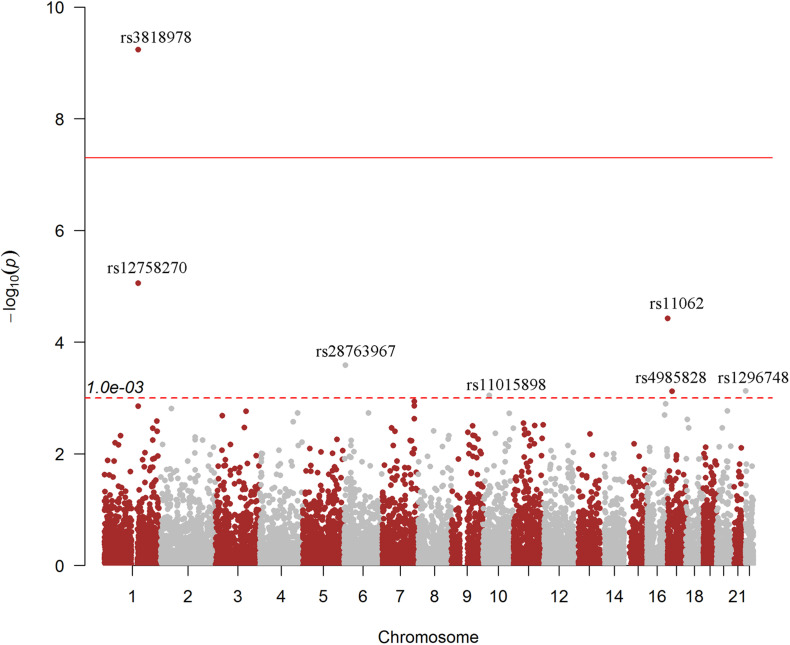
Genome-wide associations between RNA modification-associated single-nucleotide polymorphisms (RNAm-SNPs) and spontaneous coronary artery dissection (SCAD). This Manhattan plot shows the associations between RNAm-SNPs and SCAD. The *x*-axis indicates chromosome positions. The *y*-axis indicates –log_10_*p*-values of the associations. The information was extracted from the summary dataset of the SCAD genome-wide association study (GWAS) published in 2020 (Saw et al., 2020). The solid red line indicated the genome-wide significance level of 5.0 × 10^–8^, and the dotted red line indicated the suggested level of 1.0 × 10^–3^. The brown and gray color dots were used to separate them from each chromosome.

We first picked out RNAm-SNPs from the SCAD GWAS datasets based on the annotation of the nine types of RNAm-SNPs in the RMVar database (Table 1). For m^6^A modification, we found 11,464 unique RNAm-SNPs in coding and non-coding genes in the SCAD GWAS dataset. Among them, 519 (4.5%) were found to be nominally (*p* < 0.05) associated with SCAD (Table 1). Among these 519 SNPs, 213 were high-confidence RNAm-SNPs, 103 were medium-confidence RNAm-SNPs, and 203 were low-confidence RNAm-SNPs. The SNPs mainly located in exon (75), UTR (88), and intron (156). Specifically, nine RNAm-SNPs were associated with SCAD at *p* < 0.001, and rs3818978 in the 5′-UTR of *MRPS21* was significantly associated with SCAD (*p* < 5.0 × 10^–8^) (Table 2). Seven of the nine identified RNAm-SNPs were m^6^A-associated SNPs (Figure 1). The genome-wide significant SCAD m^6^A-associated SNP rs3818978 (*p* = 5.74 × 10^–10^) belongs to the medium confidence category.

**TABLE 1:** Number of each type of RNAm-SNPs examined in this study.

**RNA modification types**	**Number of SNPs found in GWAS dataset**	**Number of SNPs with *p*-value < 0.05**	**Number of SNPs with *p*-value < 0.001**
m^6^A	11,464	519	7
m^6^Am	11	1	0
m^1^A	619	24	0
m^5^C	57	1	0
m^5^U	3	0	0
m^7^G	171	14	1
2′-O-Me	4	0	0
A-to-I	377	14	1
Pseudouridine	2	0	0

*GWAS, genome-wide association study; m^2^A, N^6^-methyladenosine; RNAm-SNP, RNA modification-associated single-nucleotide polymorphism.*

**TABLE 2:** Top RNAm-SNPs associated with SCAD.

**SNP**	***p*-value**	**Type**	**CHR**	**Position**	**Gene**	**Gene region**	**Confidence level**	**Function**
rs3818978	5.74E-10	m^6^A	1	150266338	*MRPS21*	5′-UTR	MeRIP-seq:(Medium)	Functional loss
rs3818978	5.74E-10	m^6^A	1	150266338	*MRPS21*	5′-UTR	MeRIP-seq:(Medium)	Functional loss
rs12758270	8.70E-06	m^6^A	1	150446673	*RPRD2*	3′-UTR	DART-seq:(High)	Functional loss
rs12758270	8.70E-06	m^6^A	1	150446673	*RPRD2*	3′-UTR	DART-seq:(High)	Functional loss
rs28763967	2.58E-04	m^6^A	6	7581032	*DSP*	intron	Prediction:(Low)	Functional loss
rs11015898	8.91E-04	m^6^A	10	27890680	*RAB18-MKX*	Intergenic	m^6^A-Label-seq:(High)	Functional loss
rs11062	3.76E-05	m^6^A	17	1683012	*SMYD4*	3′-UTR	Prediction:(Low)	Functional gain
rs4985828	7.51E-04	m^6^A	17	20994235	*LINC01563*	Downstream	m^6^A-Label-seq:(High)	Functional loss
rs1296748	7.41E-04	m^6^A	22	17977024	*CECR2*	intron	m^6^A-Label-seq:(High)	Functional loss
rs113664950	1.83E-04	m^7^G	6	32627885	*HLA-DQB1*	exon	MeRIP-seq:(Medium)	Functional loss
rs580060	2.97E-06	A-to-I	1	150281202	*MRPS21*	3′-UTR	RNA-Seq:(High)	Functional loss
rs580060	2.97E-06	A-to-I	1	150281202	*MRPS21*	3′-UTR	RNA-Seq:(High)	Functional loss

*m^2^A, N^6^-methyladenosine; RNAm-SNP, RNA modification-associated single-nucleotide polymorphism; SCAD, spontaneous coronary artery dissection.*

We identified 171 unique m^7^G modification SNPs from the SCAD GWAS dataset, and 14 of them were nominally associated with SCAD (Table 1). The functional loss m^7^G-associated SNP rs113664950 in an exon of *HLA-DQB1* was associated with SCAD (*p* = 1.83 × 10^–4^) (Table 2). For A-to-I modification, we found 377 unique RNAm-SNPs from the SCAD GWAS dataset, and 14 of them were nominally associated with SCAD (Table 1). The functional loss A-to-I-associated SNP rs580060 in the 3′-UTR of *MRPS21* was associated with SCAD (*p* = 2.97 × 10^–6^) (Table 2). Besides, we found one m^6^Am-associated SNP, 24 m^1^A-associated SNPs, and one m^5^C-associated SNP that were nominally associated with SCAD (Table 1).

### Gene Expression Analysis

**FIGURE 2:**
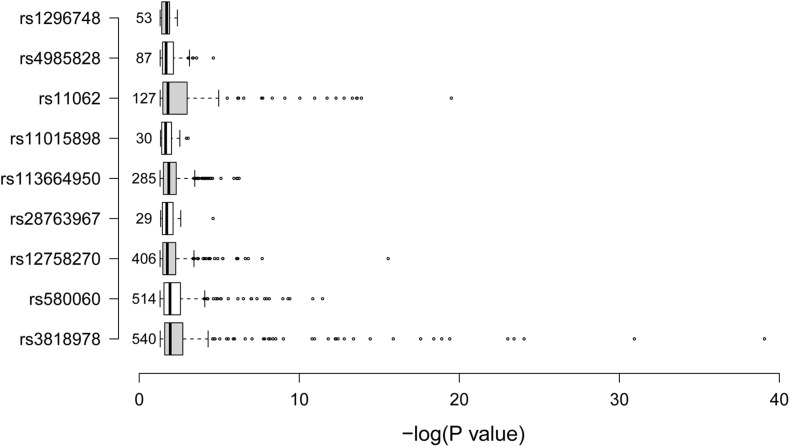
Summary of the expression quantitative loci (eQTL) signals. The boxplot summarized the –log_10_*p*-values for the eQTL signals of the nine spontaneous coronary artery dissection (SCAD)-associated RNA modification single-nucleotide polymorphism SNPs. The data were obtained from the GTEx project. The numbers on the left side of the boxes were the number of eQTL signals for the corresponding SNP.

We further tried to identify gene expressions that were associated with the nine identified SCAD-associated RNAm-SNPs (Table 2). All of these RNAm-SNPs showed eQTL effects on protein-coding genes in various types of cells or tissues from GTEx project (Figure 2). The number of eQTL signals for the nine SNPs varied (from 29 to 540). A total of 540 eQTL signals were found for rs3818978 in *MRPS21*, which was significantly associated with SCAD (*p* = 5.74 × 10^–10^). According to the HaploReg database, rs3818978 overlapped promoter histone marks in 24 tissues related to 33 bound proteins and altered four regulatory motifs. This SNP was associated with gene expression of *MRPS21* in aorta artery (*p* = 5.30 × 10^–7^) and appendage atrial tissues (*p* = 6.12 × 10^–11^) and associated with gene expression of *ADAMTSL4* in aorta artery (*p* = 8.31 × 10^–7^) and tibial artery (*p* = 1.51 × 10^–6^). The m^6^A-associated SNP rs12758270 in the 3′-UTR of *RPRD2* overlapped promoter histone marks in one tissue and enhancer histone marks in six tissues and altered two regulatory motifs. It was associated with gene expression of *ECM1* in fibroblast cells (*p* = 2.79 × 10^–5^) and esophagus mucosa (*p* = 3.69 × 10^–7^) and *ADAMTSL4* in esophagus mucosa (*p* = 9.84 × 10^–12^).

### Protein Quantitative Loci Analysis

We found 44 pQTL signals for six (rs12758270, rs28763967, rs11062, rs3818978, rs580060, and rs1296748) of the nine identified RNAm-SNPs (Table 3). A total of 42 proteins were found. Among these signals, 36 were found from the pGWAS Server (Suhre et al., 2017). The top signal was the association between rs12758270 and Extracellular matrix protein 1 (*p* = 2.66 × 10^–7^), followed by the association between rs11062 and Interleukin 4 (*p* = 6.78 × 10^–5^). Among the 44 pQTL signals, 43 signals were found for m^6^A-associated SNPs, and one signal was found for A-to-I-associated SNP rs580060, which was associated with Cystatin F (*p* = 4.69 × 10^–4^). In addition, we found that rs580060 (*p* = 1.41 × 10^–5^) and rs12758270 (*p* = 3.13 × 10^–3^) were associated with cytokine bNGF level. For lipid levels, rs3818978 was associated with plasma level of Glol (*p* = 4.62 × 10^–4^) and LDL-D (*p* = 2.63 × 10^–4^); rs580060 was associated with plasma level of XS-VLDL-PL (*p* = 2.89 × 10^–4^).

**TABLE 3:** pQTL signals for the SCAD-associated RNA modification SNPs.

**SNP**	**Associated protein**	**Coding gene**	**Allele**	**BETA**	**StdErr**	***p*-value pQTL**	**Source**
rs12758270	A disintegrin and metalloproteinase with thrombospondin motifs 4	ADAMTS4	G	0.1806	0.0693	9.29E-03	Nat Commun 2017
rs12758270	Aromatic-L-amino-acid decarboxylase	DDC	G	−0.1880	0.0692	6.70E-03	Nat Commun 2017
rs12758270	Beta-2-microglobulin	B2M	G	−0.1795	0.0640	5.16E-03	Nat Commun 2017
rs12758270	C-C motif chemokine 20	CCL20	G	0.1870	0.0694	7.14E-03	Nat Commun 2017
rs12758270	Cysteine-rich secretory protein 3	CRISP3	G	−0.2458	0.0688	3.71E-04	Nat Commun 2017
rs12758270	Extracellular matrix protein 1	ECM1	G	0.3526	0.0680	2.66E-07	Nat Commun 2017
rs12758270	Thyroid Stimulating Hormone	TSHB	G	−0.1808	0.0693	9.21E-03	Nat Commun 2017
rs28763967	A disintegrin and metalloproteinase with thrombospondin motifs 15	ADAMTS15	T	0.5301	0.1741	2.39E-03	Nat Commun 2017
rs28763967	AMP Kinase (alpha1beta1gamma1)	PRKAA1	T	0.5433	0.1840	3.22E-03	Nat Commun 2017
rs28763967	Baculoviral IAP repeat-containing protein 5	BIRC5	T	0.5718	0.1832	1.85E-03	Nat Commun 2017
rs28763967	C-C motif chemokine 21	CCL21	T	−0.4890	0.1783	6.23E-03	Nat Commun 2017
rs28763967	Chromobox protein homolog 5	CBX5	T	0.4764	0.1842	9.82E-03	Nat Commun 2017
rs28763967	Granzyme H	GZMH	T	0.4870	0.1844	8.40E-03	Nat Commun 2017
rs28763967	Hepatocyte growth factor-like protein	MST1	T	0.4982	0.1840	6.88E-03	Nat Commun 2017
rs28763967	Interleukin-17D	IL17D	T	0.5379	0.1836	3.47E-03	Nat Commun 2017
rs28763967	Ligand-dependent nuclear receptor corepressor-like protein	LCORL	T	0.5652	0.1834	2.11E-03	Nat Commun 2017
rs28763967	Phosphoglycerate kinase 1	PGK1	T	−0.5003	0.1811	5.85E-03	Nat Commun 2017
rs28763967	Ras GTPase-activating protein 1	RASA1	T	0.5732	0.1831	1.80E-03	Nat Commun 2017
rs28763967	RNA-binding protein 39	RBM39	T	0.4790	0.1839	9.33E-03	Nat Commun 2017
rs28763967	Sialoadhesin	SIGLEC1	T	0.6622	0.1820	2.89E-04	Nat Commun 2017
rs11062	Beta-2-microglobulin	B2M	A	−0.1252	0.0435	4.10E-03	Nat Commun 2017
rs11062	C-C motif chemokine 14	CCL14	A	−0.1282	0.0458	5.24E-03	Nat Commun 2017
rs11062	Elongation factor 1-beta	EEF1B2	A	0.1304	0.0467	5.33E-03	Nat Commun 2017
rs11062	Fatty acid-binding protein, heart	FABP3	A	−0.1178	0.0447	8.60E-03	Nat Commun 2017
rs11062	Glycylpeptide N-tetradecanoyltransferase 1	NMT1	A	0.1269	0.0466	6.62E-03	Nat Commun 2017
rs11062	Granulysin	GNLY	A	−0.1295	0.0464	5.37E-03	Nat Commun 2017
rs11062	Granzyme A	GZMA	A	−0.1219	0.0470	9.59E-03	Nat Commun 2017
rs11062	Interleukin-4	IL4	A	0.1859	0.0465	6.78E-05	Nat Commun 2017
rs11062	Peptidyl-prolyl *cis-trans* isomerase E	PPIE	A	0.1324	0.0466	4.57E-03	Nat Commun 2017
rs11062	Platelet glycoprotein VI	GP6	A	0.1338	0.0468	4.30E-03	Nat Commun 2017
rs11062	Receptor-type tyrosine-protein kinase FLT3	FLT3	A	0.1226	0.0466	8.68E-03	Nat Commun 2017
rs11062	Sialic acid-binding Ig-like lectin 14	SIGLEC14	A	−0.1431	0.0471	2.46E-03	Nat Commun 2017
rs11062	S-phase kinase-associated protein 1	SKP1	A	0.1320	0.0471	5.13E-03	Nat Commun 2017
rs11062	Trefoil factor 3	TFF3	A	−0.1174	0.0445	8.53E-03	Nat Commun 2017
rs11062	Tumor necrosis factor-inducible gene 6 protein	TNFAIP6	A	−0.1278	0.0449	4.48E-03	Nat Commun 2017
rs11062	Ubiquitin-conjugating enzyme E2 L3	UBE2L3	A	0.1218	0.0467	9.31E-03	Nat Commun 2017
rs3818978	C-C motif chemokine 14	CCL14	A	−0.0847	0.0256	9.38E-04	Nature 2018
rs580060	Cystatin-F	CST7	A	0.0885	0.0253	4.69E-04	Nature 2018
rs1296748	Retinoic acid receptor responder protein 1	RARRES1	T	−0.1087	0.0313	5.15E-04	Nature 2018
rs3818978	C-C motif chemokine 22	CCL2	T	−0.1053	0.0408	9.86E-03	PLoS Genet 2017
rs3818978	C-X-C motif chemokine 16	CXCL16	T	−0.1078	0.0408	8.24E-03	PLoS Genet 2017
rs3818978	Dickkopf-related protein 1	DKK1	T	−0.1200	0.0408	3.27E-03	PLoS Genet 2017
rs3818978	Heparin-binding EGF-like growth factor	HBEGF	T	−0.1162	0.0408	4.40E-03	PLoS Genet 2017
rs3818978	Epidermal growth factor	EGF	T	−0.1255	0.0408	2.10E-03	PLoS Genet 2017

*pQTL, protein quantitative loci; SCAD, spontaneous coronary artery dissection; SNP, single-nucleotide polymorphism.*

### Functional Enrichment Analysis

**FIGURE 3:**
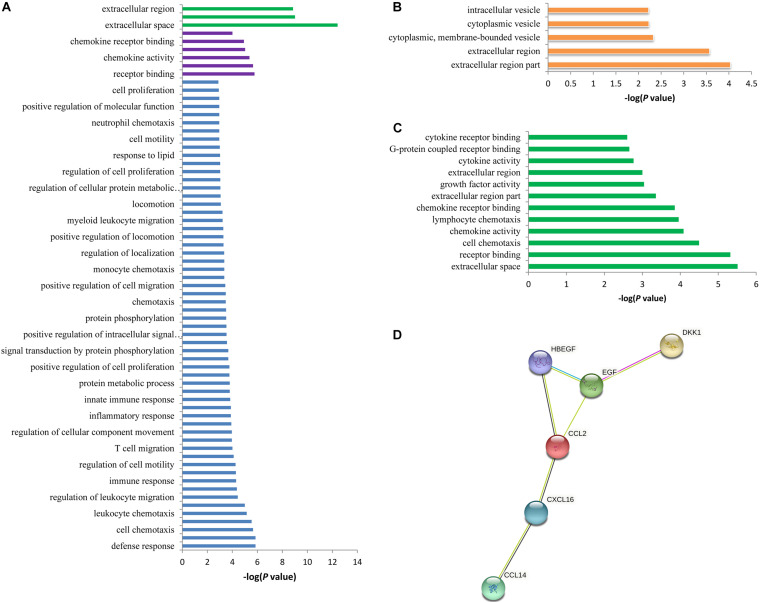
Functional annotation enrichment analysis of the spontaneous coronary artery dissection (SCAD)-related proteins. **(A)** There were 68 statistically significant Gene Ontology (GO) terms that were enriched in the SCAD-related proteins. **(B)** The seven proteins (ADAMTS4, DDC, B2M, CCL20, CRISP3, ECM1, and TSHB) that may be affected by rs12758270 were enriched in five GO terms. **(C)** The six proteins (CCL2, CXCL16, DKK1, HBEGF, EGF, and CCL14) that may be affected by rs3818978 were enriched in 12 GO terms. **(D)** Protein–protein interactions between the six proteins that may be affected by rs3818978.

The 42 associated proteins were enriched in 68 Gene Ontology (GO) terms, including cellular components such as extracellular space (*p* = 3.80 × 10^–13^) and extracellular region (*p* = 1.40 × 10^–9^); biological process such as defense response (*p* = 1.40 × 10^–6^) and lymphocyte migration (*p* = 1.40 × 10^–6^); molecular function such as receptor binding (*p* = 1.70 × 10^–6^) and cytokine receptor binding (*p* = 2.20 × 10^–6^); and so on (Figure 3A). Plasma levels of seven proteins coded by *ADAMTS4*, *DDC*, *B2M*, *CCL20*, *CRISP3*, *ECM1*, and *TSHB* were affected by rs12758270 (Table 3). These proteins were enriched in cellular component such as extracellular region (*p* = 9.30 × 10^–5^) and cytoplasmic vesicle (*p* = 5.90 × 10^–3^) (Figure 3B).

The genome-wide significant SCAD-associated m^6^A SNP rs3818978 was associated with the plasma levels of CCL2, CXCL16, DKK1, HBEGF, EGF, and CCL14 (Table 3). These six proteins were enriched in specific biological processes (e.g., cell chemotaxis and lymphocyte chemotaxis), cellular component (e.g., extracellular space, extracellular region part, and extracellular region), and molecular function (e.g., receptor binding, chemokine activity, chemokine receptor binding, growth factor activity, and cytokine activity) (Figure 3C). These six proteins interacted directly with each other according to the STRING database (Figure 3D).

## Discussion

This study showed that SCAD-associated SNPs identified in GWAS were related to RNA modification. These SNPs showed *cis*-acting eQTL effects in various types of cells and tissues, and some of them were found to be associated with circulating protein levels. Moreover, the associated proteins were enriched in specific functional annotation terms.

Increasing evidence has shown that m^6^A methylation is critical in gene expression regulation, as it modulates RNA processing such as nuclear export, translatability, splicing, and stability (Li et al., 2018; Visvanathan and Somasundaram, 2018). Variations in the methylation target sites can interrupt the modification and then disturb gene expression regulations (Zheng et al., 2018). Therefore, identification of RNAm-SNPs in diseases is a way to ascertain causal variants and therefore helpful for the interpretation of the GWAS findings. In the present study, we identified many SCAD-associated RNAm-SNPs and showed that these kinds of SNPs may have impacts on gene expression, including mRNA levels and protein levels. The findings suggested that RNA modification may play a role in SCAD.

One of the genome-wide significant RNAm-SNPs, rs3818978, has not been discussed in the SCAD GWAS paper. This m^6^A-associated SNP was close to the reported SCAD-associated SNP rs12740679 (4 Kb upstream of gene *MRPS21*). RNAm-SNP rs3818978 locates in the 5′-UTR of *MRPS21*. The function of *MRPS21* gene has been less known. According our analysis, rs3818978 was associated with the expression of *MRPS21* and *ADAMTSL4* in the artery aorta and associated with the plasma level of several proteins with specific functions. In *MRPS21*, there was another SNP identified in this study, the A-to-I modification SNP rs580060, which locates in the 3′-UTR of *MRPS21*. This SNP was also associated with the expression of *ADAMTSL4* in the artery aorta and associated with the plasma level of Cystatin F. *ADAMTSL4* codes an extracellular matrix protein. The extracellular matrix protein binds to fibrillin-1 to form microfibrils in the matrix (Hubmacher and Apte, 2015). The original SCAD GWAS has demonstrated that *ADAMTSL4* gene was expressed in the interarterial layer and vascular smooth muscle cells. It was suggested that *ADAMTSL4* deficiency was involved in arterial fragility (Saw et al., 2020). The present study identified two RNA modification SNPs that may affect *ADAMTSL4* expression as potential functional variants for SCAD.

In addition to the genome-wide significant signals, we found several RNAm-SNPs that did not reach the genome-wide significance threshold of 5.0 × 10^–8^ for SCAD. For example, the m^6^A-associated SNP rs12758270 that locates in the 3′-UTR of *RPRD2* did not reach the genome-wide significant level for SCAD. Then, it has been ignored in the original GWAS. In the present study, we found that rs12758270 was associated with gene expression of *ECM1* and *ADAMTSL4* and associated with plasma levels of ADAMTS4, DDC, B2M, CCL20, CRISP3, ECM1, and TSHB, which have specific biological functions. Therefore, these RNAm-SNPs may also be potential functional candidates for SCAD.

The RNAm-SNPs could disturb RNA modification, and then the mRNA expression levels were changed and consequently affect SCAD risk. But further evidence is needed to prove that gene expression affected by these RNAm-SNPs was associated with SCAD risk. According to the result of a transcriptome sequencing study (GSE153434), *DSP* (fold change = 0.327, *p* = 2.30 × 10^–4^), *MKX* (fold change = 0.391, *p* = 3.83 × 10^–8^), and *SERPINF1* (fold change = 0.066, *p* = 2.26 × 10^–15^) were differentially expressed between Stanford type A aortic dissection patients and controls in ascending aortic tissues (Zhou et al., 2020). *SERPINF1* expression level was associated with rs11062 (m^6^A-associated SNP locates in the 3′-UTR of *SMYD4*) in transformed fibroblast cells, left ventricle, subcutaneous adipose, atrial appendage, and esophagus mucosa tissues. In addition, our analysis showed that RNAm-SNPs affected genes involved in specific biological functions, which were highly related to SCAD. SCAD is mediated by inflammasome activation, which exacerbates the secretion of pro-inflammatory cytokines, chemokines, and matrix metalloproteinases. The dysregulation of extracellular matrix results in progressive smooth muscle cell depletion and inflammation, leading to SCAD (Mayer et al., 2020; Shen et al., 2020). Therefore, gene expressions affected by the RNAm-SNPs play important roles in SCAD. This evidence may suggest that the identified RNAm-SNPs may participate in the pathogenesis of SCAD by altering RNA modification.

This study has some potential limitations. First, the sample size of the SCAD GWAS was still relatively small. Second, the m^6^A SNP set was large, but data for other types of RNA modification were still very rare. Third, although we identified associations between SCAD-associated SNPs and gene expression and plasma protein levels, the associations between the gene expression and plasma protein levels and SCAD have not been examined. Finally, the functions of these SNPs and the effects of modification of the detected genes on SCAD have not been examined experimentally. More evidence is needed to prove that these SNPs may indeed participate in the pathogenesis of SCAD by altering RNA modification. Further experiments determining the functions in related cells (e.g., vascular smooth muscle cells) are needed.

## Conclusion

This study found SCAD-associated RNAm-SNPs and therefore suggested that RNA modification may play important roles in SCAD. The findings increased our understanding on the associations identified in the SCAD GWAS. These kinds of SNPs in susceptibility genes of SCAD may work by regulating gene expression, including mRNA levels and protein levels. No previous study has shown the relationship between RNA modification and SCAD. Therefore, this study may add valuable clues for further understanding of functional mechanisms underlying the development of SCAD.

## Data Availability Statement

The original contributions presented in the study are included in the article/supplementary material, further inquiries can be directed to the corresponding author.

## Author Contributions

TC, MT, and LC contributed to the conceptualization. TC contributed to data curation and writing of the original draft. MT contributed to the formal analysis. TC, MT, XY, ZQ, and XL contributed to the investigation. TC, MT, and XL contributed to the methodology. TC and MT contributed to the visualization. MT and LC contributed to the writing, review, and editing. All authors contributed to the article and approved the submitted version.

## Conflict of Interest

The authors declare that the research was conducted in the absence of any commercial or financial relationships that could be construed as a potential conflict of interest.

## Footnotes

1 http://rmvar.renlab.org/download.html

2 http://archive.broadinstitute.org/mammals/haploreg/haploreg.php

3 http://regulome.stanford.edu/

4 http://www.gtexportal.org

5 http://metabolomics.helmholtz-muenchen.de/pgwas/index.php?task=download

6 http://www.phpc.cam.ac.uk/ceu/proteins/

7 https://zenodo.org/record/264128#.X3u5ga95uUk

8 http://www.computationalmedicine.fi/data#Cytokine_GWAS
